# Supplementary material for: The Construction and Exploration of a Comprehensive MicroRNA Centered Regulatory Network in Foxtail Millet (Setaria italica L.)
Source: Front Plant Sci. 2022 May 6;13:848474. doi: 10.3389/fpls.2022.848474 (PMC9121102; doi:10.3389/fpls.2022.848474)
Supplement: Supplementary file 3 [file Data_Sheet_3.zip › Sit-miR394_Seita.9G235000.1_568_TPlot.pdf]

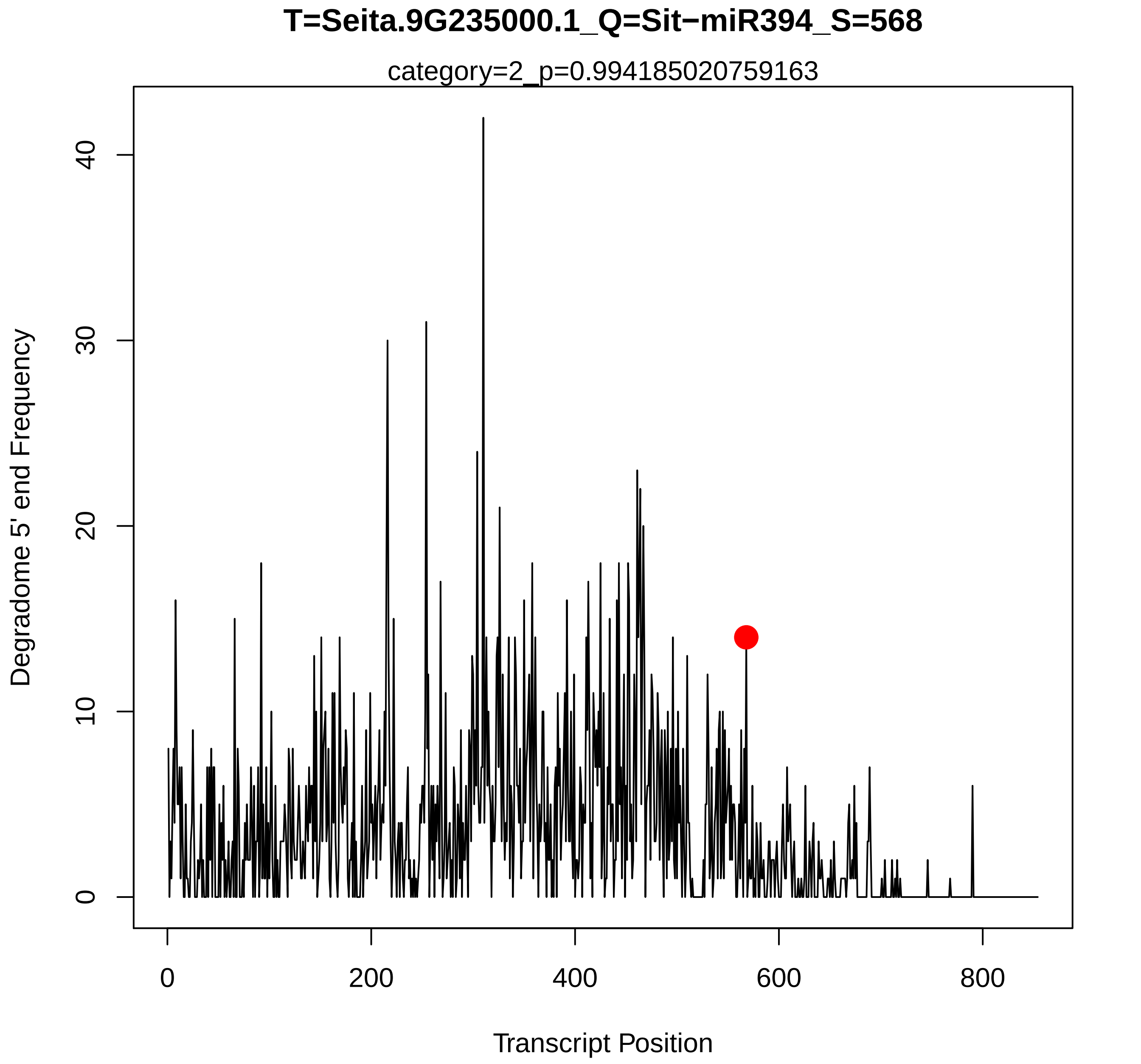

T=Seita.9G235000.1_Q=Sit−miR394_S=568
category=2_p=0.994185020759163
Degradome 5' end Frequency
Transcript Position
0
10
20
30
40
0
200
400
600
800
